# Supplementary material for: Deep generative modeling of transcriptional dynamics for RNA velocity analysis in single cells
Source: Nat Methods. 2023 Sep 21;21(1):50–9. doi: 10.1038/s41592-023-01994-w (PMC10776389; doi:10.1038/s41592-023-01994-w)
Supplement: Supplementary file 1 — Supplementary Table 1, Supplementary Figs. 1–12 and Supplementary Notes 1–4. [file 41592_2023_1994_MOESM1_ESM.pdf]

nature methods

Article https://doi.org/10.1038/s41592-023-01994-w

Deep generative modeling of transcriptional dynamics for RNA velocity analysis in single cells

In the format provided by the authors and unedited

Contents

Supplementary Figures 2

Supplementary Note 1 9
PBMC case study 9

Supplementary Note 2 11
Dentate gyrus case study 11

Supplementary Note 3 13
Related work 13

Supplementary Note 4 15
Modeling considerations, limitations, and future directions 15

Supplementary Tables 19

Supplementary Figures

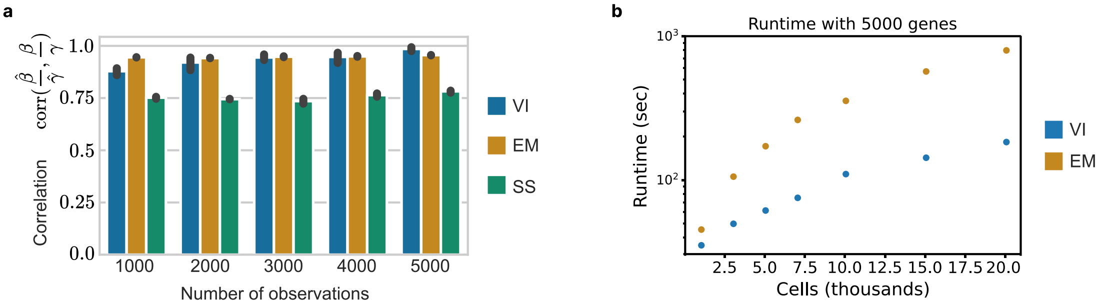

Supplementary Figure 1: Benchmarking veloVI. a, Correlation between the estimated ratio of splicing and degradation rates, and ground truth on simulated data using veloVI (VI, blue), the *EM model* (EM, orange), steady-state model (SS, green). For each number of observations, 10 datasets were generated, and the average is shown with the corresponding 95% confidence interval. **b**, Runtime comparison between veloVI (VI, orange) and the *EM model* (EM, blue). The *EM model* was run on an Intel(R) Core(TM) i9-10900K CPU @ 3.70GHz with 8 cores, veloVI on an Nvidia RTX3090 GPU.

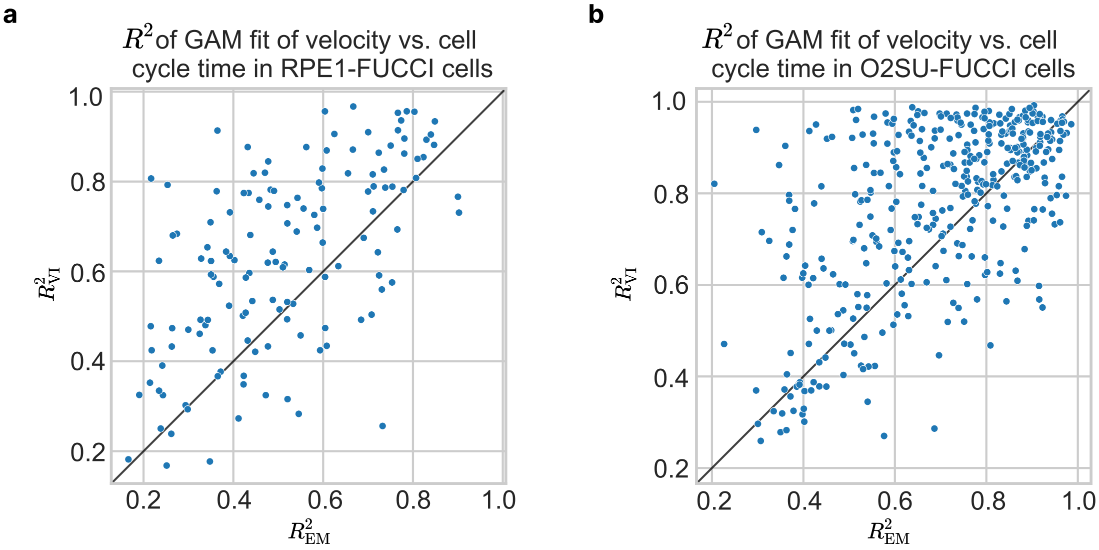

Supplementary Figure 2: Velocity evaluation for the cell cycle of RPE1- and U2OS-FUCCI cells a, The R^2 of the fitted GAMs using veloVI vs. the *EM model* in RPE1-FUCCI cells. For each gene, one GAM approximated the relationship between the inferred velocities and a cell cycle score. **b**, Same as **a** but using the U2OS-cells.

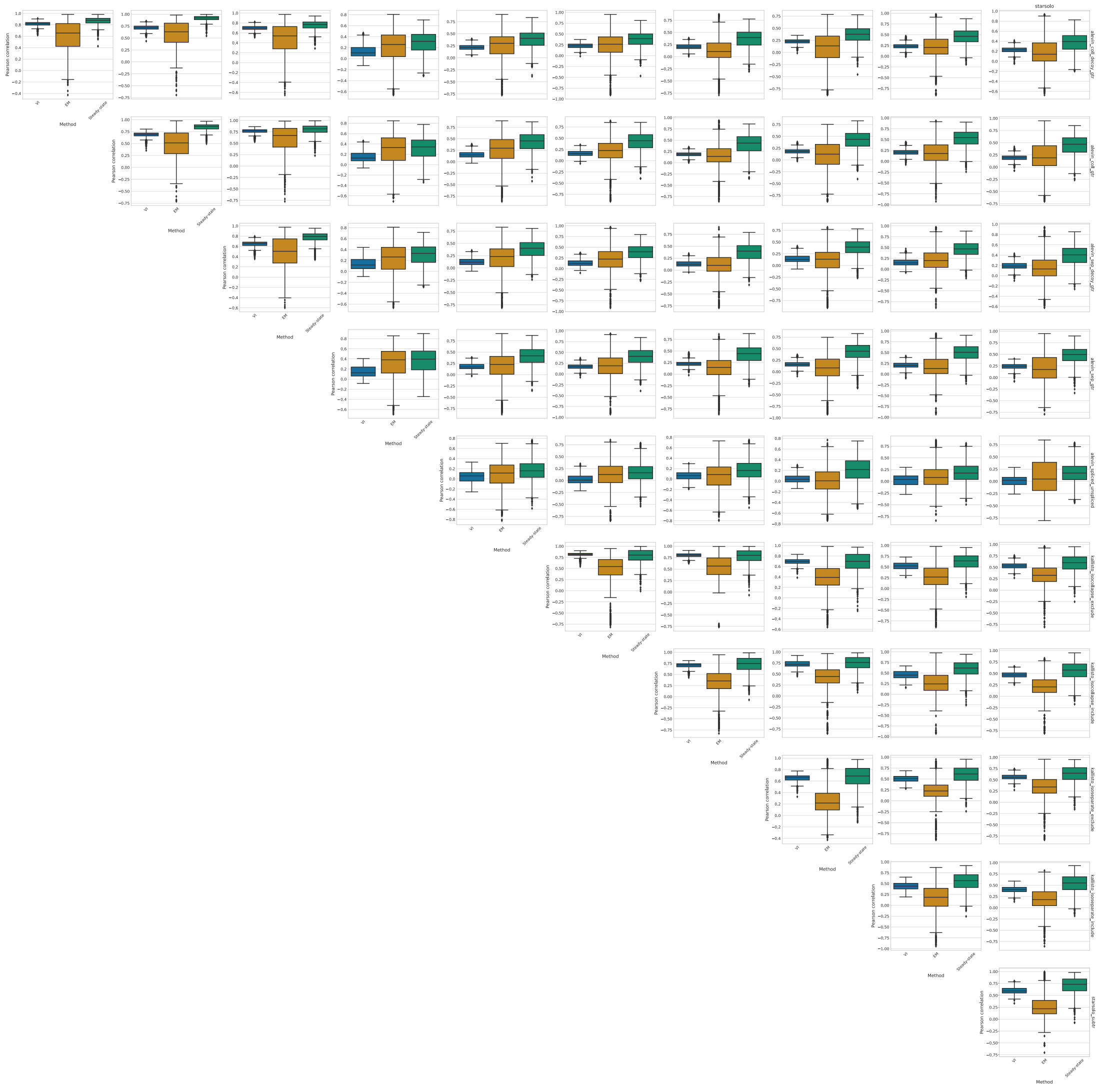

Supplementary Figure 3: Preprocessing robustness for the dentate gyrus dataset. Pair-wise correlations of velocity between pre-processing protocols. Velocities are inferred using veloVI, the EM model, or the steady-state model. For each pair of quantification algorithms and inference method, the correlation between the two inferred velocities for a cell are correlated. Box plots indicate the median (center line), interquartile range (hinges), and whiskers at 1.5x interquartile range ($N = 2914$ cells each).

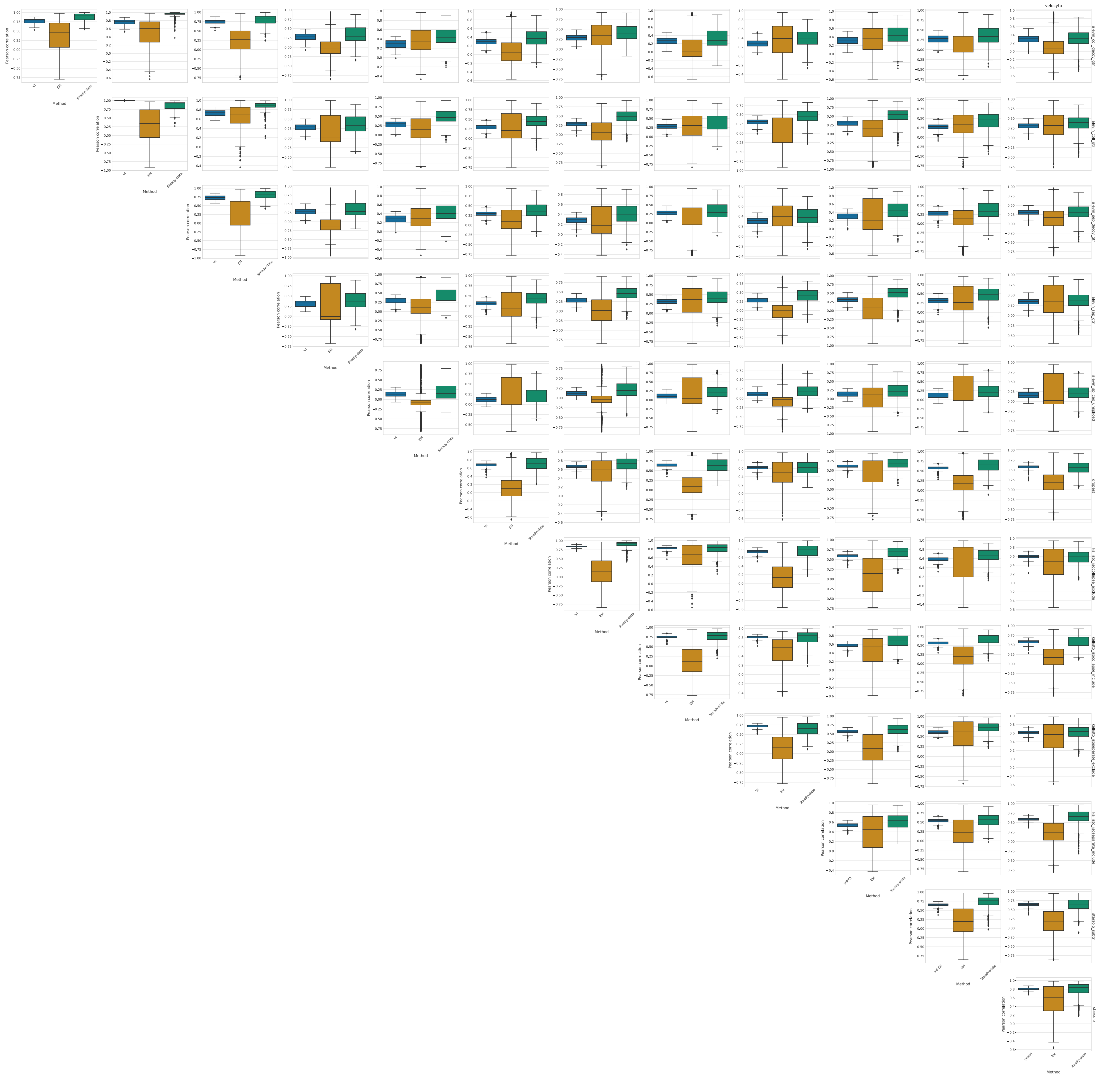

Supplementary Figure 4: Preprocessing robustness for the old brain dataset. Pair-wise correlations of velocity between pre-processing protocols. Velocities are inferred using veloVI, the EM model, or the steady-state model. For each pair of quantification algorithms and inference method, the correlation between the two inferred velocities for a cell are correlated. Box plots indicate the median (center line), interquartile range (hinges), and whiskers at 1.5x interquartile range ($N = 1823$ cells each).

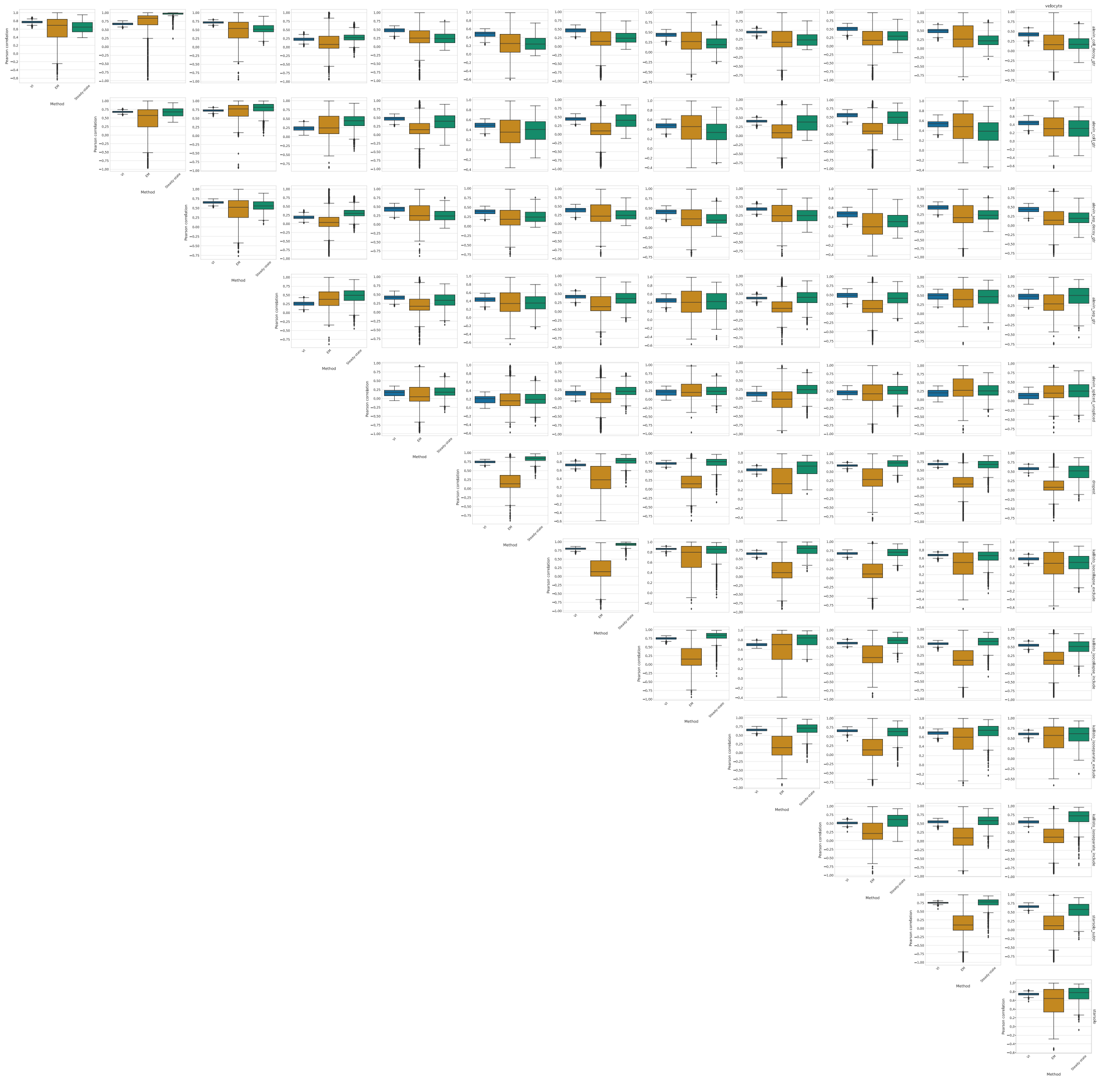

Supplementary Figure 5: Preprocessing robustness for the pancreas dataset. Pair-wise correlations of velocity between pre-processing protocols. Velocities are inferred using veloVI, the EM model, or the steady-state model. For each pair of quantification algorithms and inference method, the correlation between the two inferred velocities for a cell are correlated. Box plots indicate the median (center line), interquartile range (hinges), and whiskers at 1.5x interquartile range ($N = 3696$ cells each).

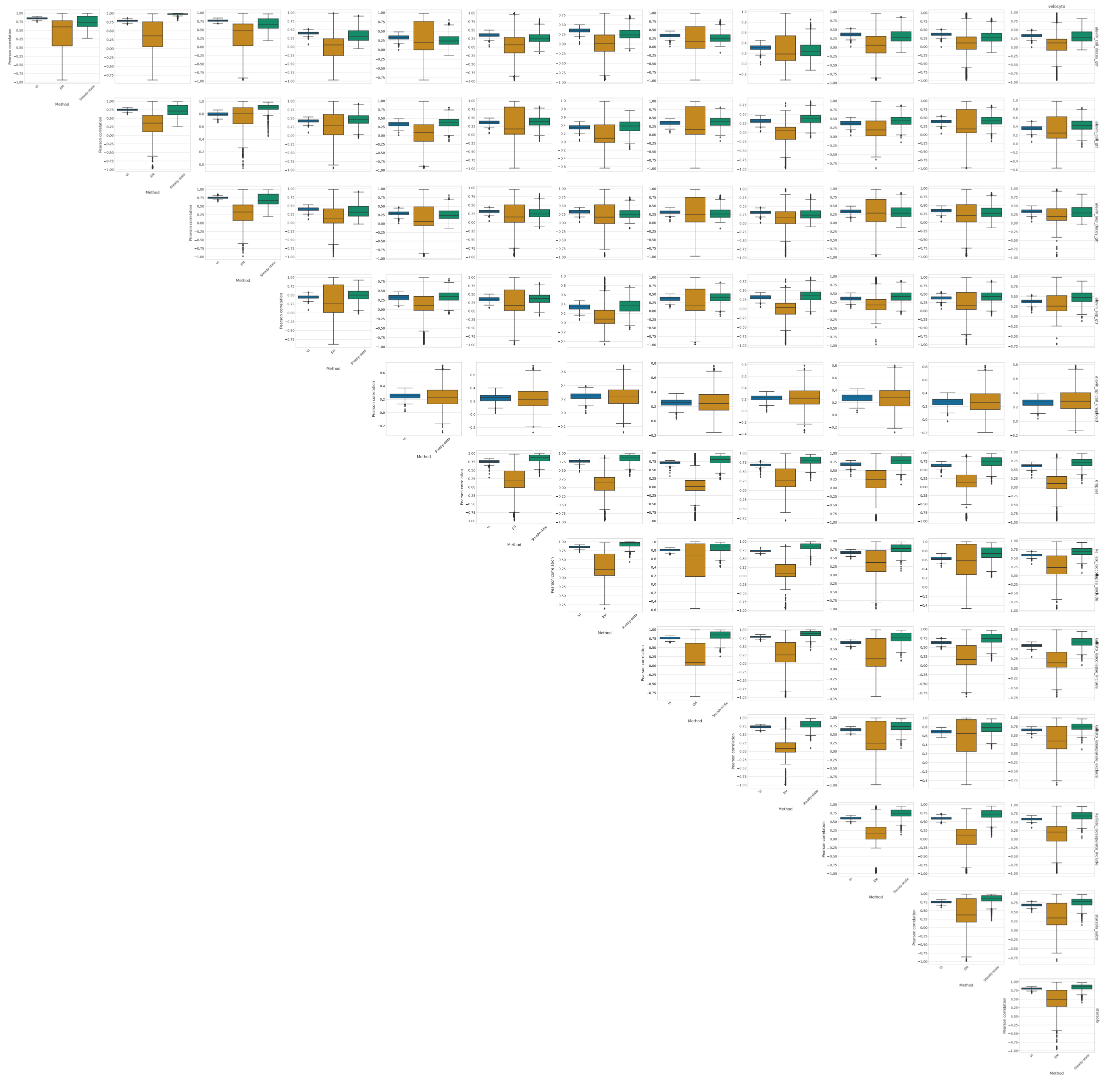

Supplementary Figure 6: Preprocessing robustness for the PFC dataset. Pair-wise correlations of velocity between pre-processing protocols. Velocities are inferred using veloVI, the EM model, or the steady-state model. For each pair of quantification algorithms and each inference method, the correlation between the two inferred velocities for a cell are correlated. Box plots indicate the median (center line), interquartile range (hinges), and whiskers at 1.5x interquartile range ($N = 1267$ cells each).

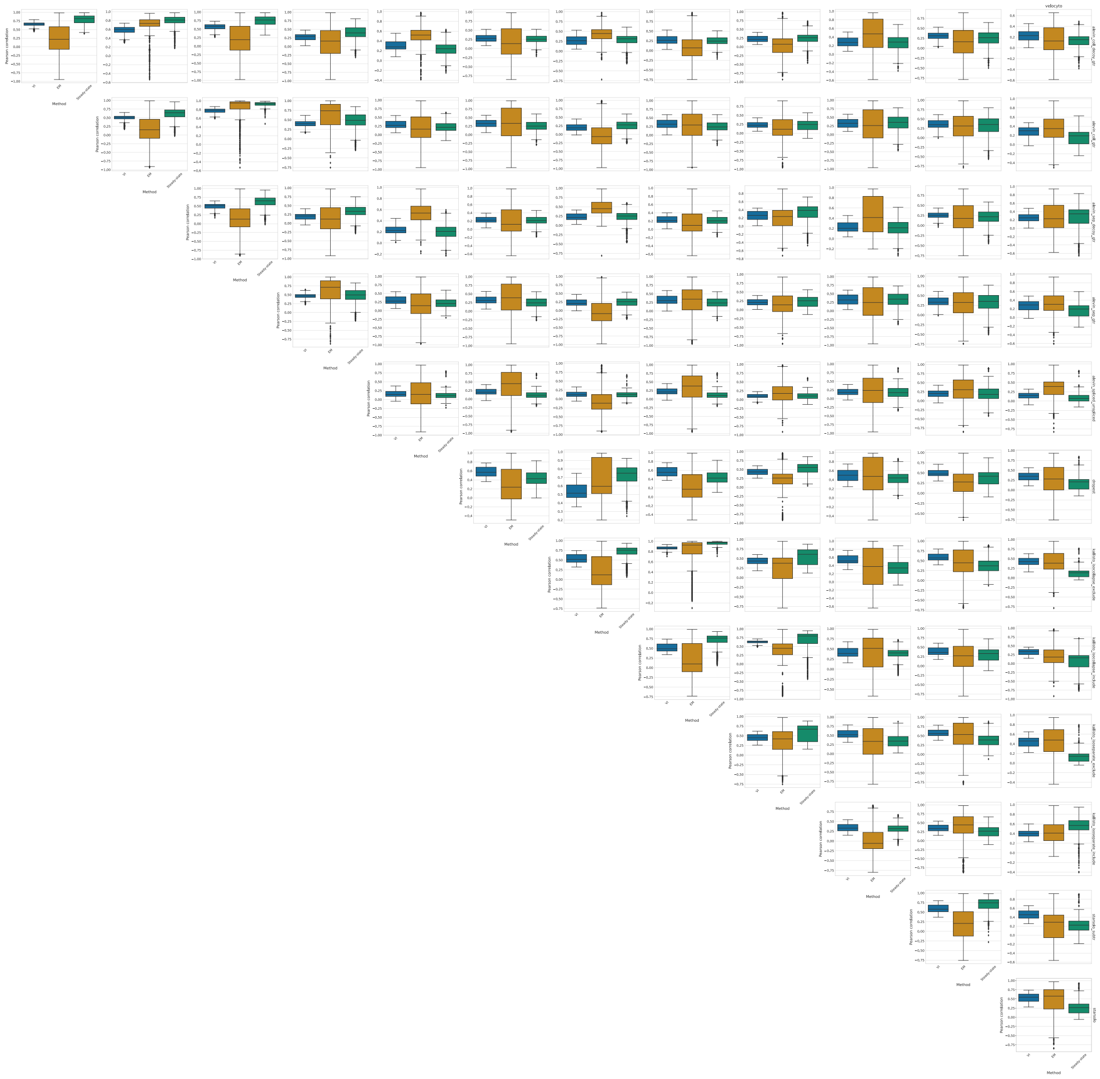

Supplementary Figure 7: Preprocessing robustness for the spermatogenesis dataset. Pair-wise correlations of velocity between pre-processing protocols. Velocities are inferred using veloVI, the EM model, or the steady-state model. For each pair of quantification algorithms and inference method, the correlation between the two inferred velocities for a cell are correlated. Box plots indicate the median (center line), interquartile range (hinges), and whiskers at 1.5x interquartile range ($N = 1829$ cells each).

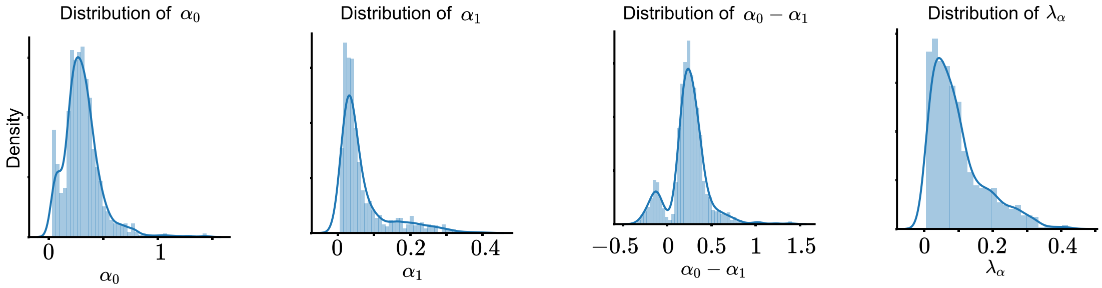

Supplementary Figure 8: Time-dependent transcription rate. Distribution of inferred parameters of time-dependent transcription rate.

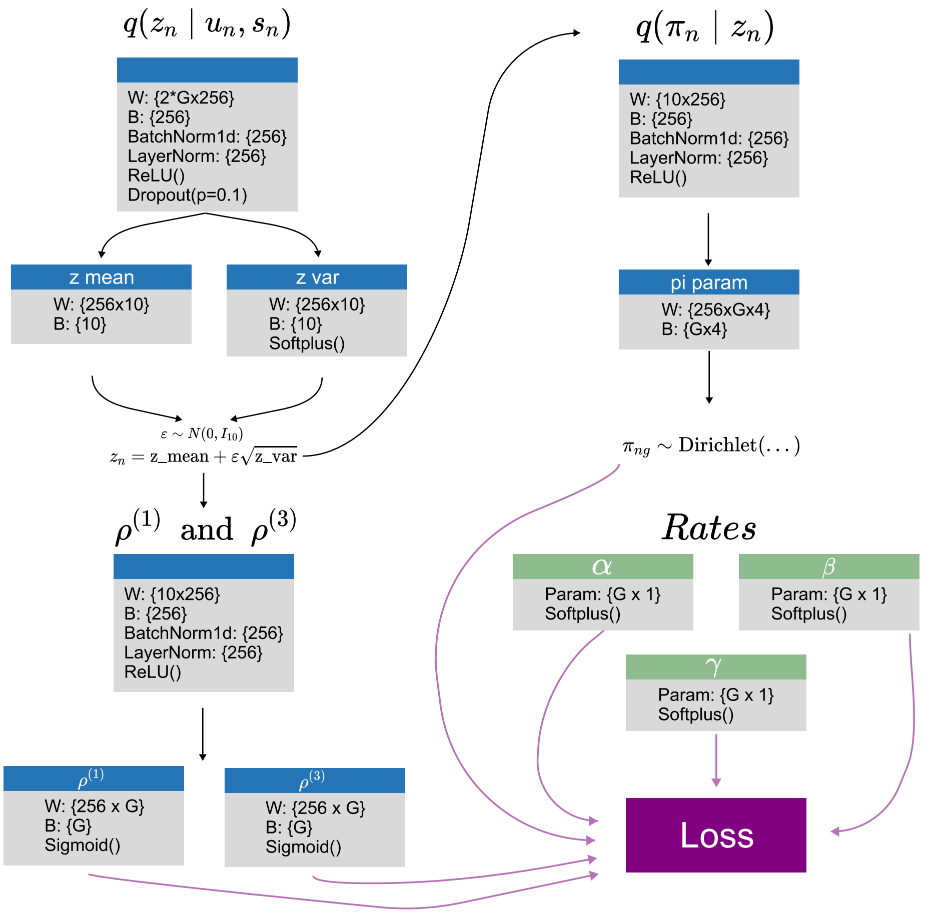

Supplementary Figure 9: Overview of veloVI architecture. W denotes a weight matrix, B denotes a bias term. Param denotes a learnable parameter.

Supplementary Note 1

PBMC case study

Here, we focus on applying veloVI to a dataset of peripheral blood mononuclear cells [1, 2] (Supplementary Figure 10a). This public dataset from 10x Genomics was processed with Kallisto Bustools [3] and automatically annotated via totalVI [4] using the Seurat v3 CITE-seq PBMC dataset [1, 2] as a reference. As the dataset contains fully mature cell types, we expect the cells to be in steady-state with respect to RNA metabolism. Hence, we posit that RNA velocity cannot be used to gain insight into cell type transitions in this dataset.

After estimating velocities with veloVI and visualizing using popular techniques (Supplementary Figure 10a), we quantified the corresponding extrinsic uncertainties. The overall increased extrinsic uncertainty in the cluster labelled as "other", as well as the substantial distribution mass of every other cell type away from the origin suggests that an analysis based RNA velocity is not suitable (Supplementary Figure 10b, c).

To assess the coherence of velocity estimates, we can study our proposed velocity coherence score. The velocity coherence in monocytes, B, and natural killer cells is close to symmetric around zero (Supplementary Figure 10d). Consequently, the inferred models include (approximately) equal number of cases in which the inferred velocity and empirical displacement of a cell agree and disagree. This result also undermines the use of RNA velocity on this dataset as there is no clear set of genes that agrees with the consensus directionality.

Finally, we calculated the permutation score to identify genes sensitive to changes in the abundance of unspliced and spliced mRNA. In the case of genes such as *TNFIAP6* and *CTSL*, the cell-type-specific permutation score is low (Supplementary Figure 10e). Consequently, these genes likely add noise to the directionality as they do not provide a signal displaying transient dynamics. This metric-based decision is confirmed by the corresponding phase portrait themselves as they do not exhibit the required (partial) almond shape translating, under the given model assumptions, to induction and repression states (Supplementary Figure 10e).

Conversely to genes ill-suited for RNA velocity analysis, we can focus on candidate genes scoring a high permutation score. The genes assigned a high permutation score across cell types included *EPHB1* and *CLEC4C*. However, the high permutation score is solely observed in dendritic cells (Supplementary Figure 10e). Again, studying the corresponding phase portraits, we can conclude that the score is likely the result of dendritic cells forming an outlier cluster as the phase portraits show discontinuity.

Taking all observations and metrics into consideration, we can conclude that caution is warranted if the dataset of PBMCs were to be analyzed using RNA velocity. This conclusion aligns with the biological ground truth that these cell types are in steady-state.

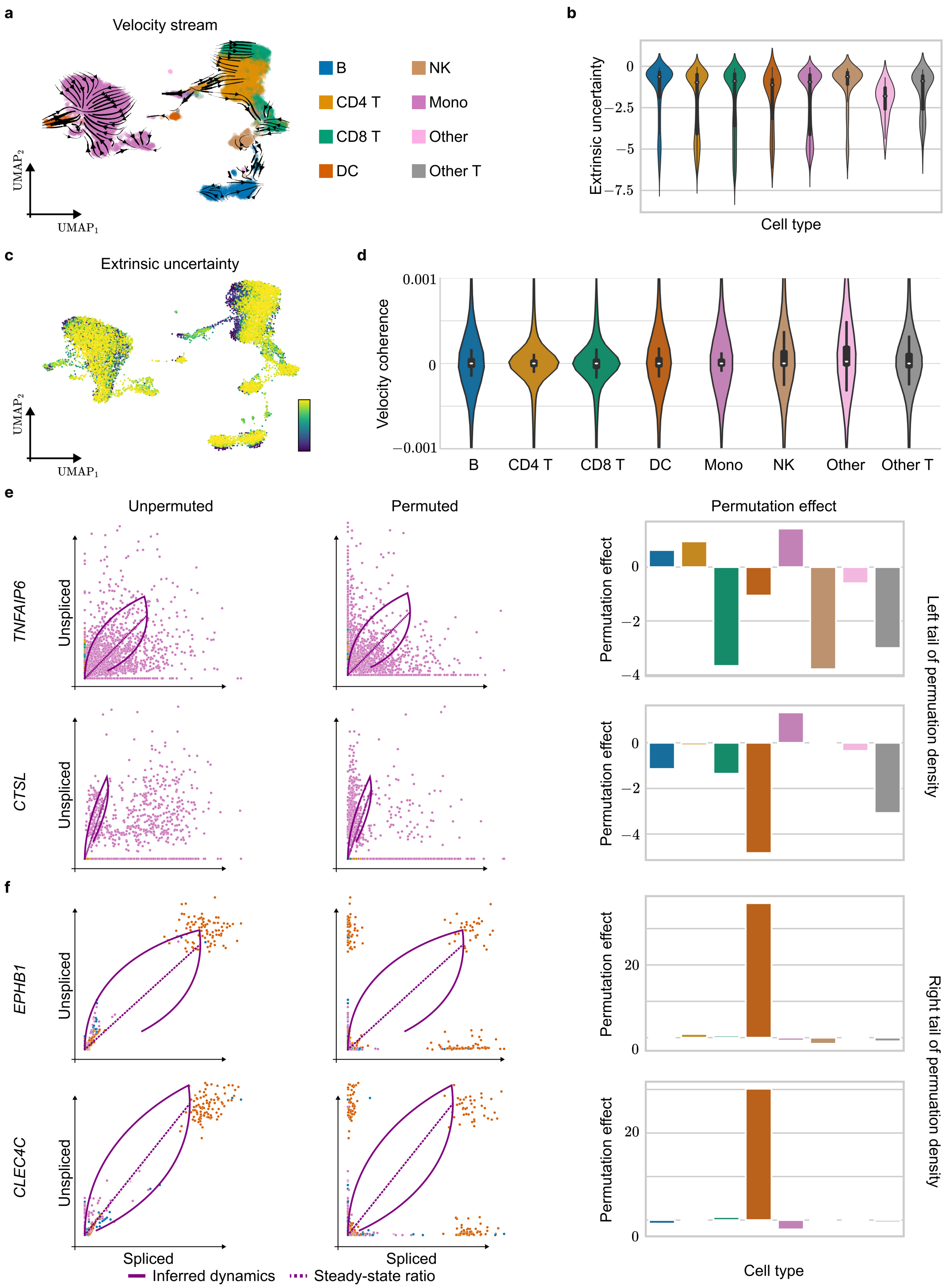

Supplementary Figure 10: Analysis of peripheral blood mononuclear cells. a. Two-dimension UMAP projection of PBMC dataset consisting of approximately 12,000 cells with inferred velocity stream projection. Clusters are colored by cell type (dendritic cells (DC), monocytes (Mono), natural killer (NK)). **b.** The corresponding extrinsic uncertainty resolved each cell type. Colors are the same as in panel **a**. Box plots indicate the median (center line), interquartile range (hinges), and whiskers at 1.5x interquartile range ($N = 1144$ B cells, $N = 3032$ CD4 T cells, $N = 2005$ CD8 T cells, $N = 240$ DC cells, $N = 4898$ monocytes, $N = 478$ natural killer cells, $N = 122$ other T cells, $N = 31$ other cells). **c.** The two-dimensional UMAP projection colored by the extrinsic uncertainty.**d.** The velocity coherence in each cell type. **e.** The phase portrait of the real., unpermuated data (left), permuted data (middle), and result cell-type specific permutation score. Results are shown for Genes *TNFIAP6* (top) and *CTSL* (bottom). Colors are according to cell type in panel a. **f.** Same as panel e but for *EPHB1* (top) and *CLEC4C* (bottom).

Supplementary Note 2

Dentate gyrus case study

The analysis of data using RNA velocity currently usually consists in inferring velocities and projecting them onto a low-dimensional representation of the data (e.g., UMAP [6]). To highlight and showcase how our proposed veloVI method can both infer RNA velocity and aid in understanding its applicability and result, here, we conduct a case study on a dataset of dentate gyrus neurogenesis [5]. This dataset is expected to be a positive control with putative transient dynamics.

As a first step, velocities are inferred using veloVI and the corresponding stream projected onto a two-dimensional UMAP embedding of the data (Supplementary Figure 11a). Here, we observe a flow from granule mature to immature cells which is putatively incorrect according to biological ground truth (granule immature to mature cells). While the intrinsic and extrinsic uncertainties are lower for neuroblast and granule immature cells, both uncertainties are elevated in the cluster of granule mature cells (Supplementary Figure 11b, c).

In addition, the velocity coherence for a given cell type can be quantified. In case of the granule mature cells, the metric is, to a majority, positive (Supplementary Figure 11d). The positivity, thus, shows that both velocity and empirical displacement under the induced transition matrix agree. However, as the mean of the distribution is close to zero, and both the extrinsic and intrinsic uncertainties are high, velocity estimates are most likely not robustly estimated.

Similarly to the granule mature cells, we observe increased uncertainties in the clusters of endothelial, oligodendrocyte precursor (OP), myelinating oligodendrocytes (OL), and microglia cells. Additionally, the velocity coherence in these cell types is mostly zero and its distribution symmetric around the origin (Supplementary Figure 11d). Together with the fact that these cell types form distinct, disconnected clusters, we can conclude that these cell types may need to be excluded from the RNA velocity analysis. One reason to exclude these cell types is that their dynamics are distinct from the remaining dataset, or corresponding transient cell populations have not been observed. In phase portraits these cases may manifest themselves as trajectories deviating from the expected almond shape or outlier clusters.

Another tool to assess the applicability of RNA velocity to the given dataset as a whole, as well as individual genes is the permutation score. Genes scoring a large permutation score across different cell types are likely to show transient dynamics. Studying the distribution of the maximum permutation score over cell types shows that granule immature and neuroblast cells are most sensitive to permutation (Supplementary Figure 11e). This result suggests that the two populations are transient which aligns with the underlying known dynamics in dentate gyrus neurogenesis.

At the level of a single gene, the permutation score reveals that in *Tmsb10*, for example, four cell types (neuroblast, granule immature, endothelial, and GABA) are sensitive to permutation (Supplementary Figure 11e). Although the fit is confounded by the endothelial cluster, it is correctly inferred for the neuroblast to granule immature lineage. Similarly, as endothelial cells are not contained in the neuroblast to granule immature lineage, this result also shows that *Tmsb10* contains multiple kinetics. Consequently, these observations show that a single gene-specific model is inappropriate, or that only granule lineage cell types should be considered for this gene.

Genes inappropriate for RNA velocity analysis can be identified similarly based on the permutation score. The low permutation scores for each cell type in *Eed*, for example, show that it should not be considered for RNA velocity analysis in the first place (Supplementary Figure 11f). The low cell-type-specific scores stem from the non-transient and noisy nature of the unspliced and spliced abundance. Consequently, they do not reflect the expected almond shape given the model assumptions, and yield the parameter inference non-robust.

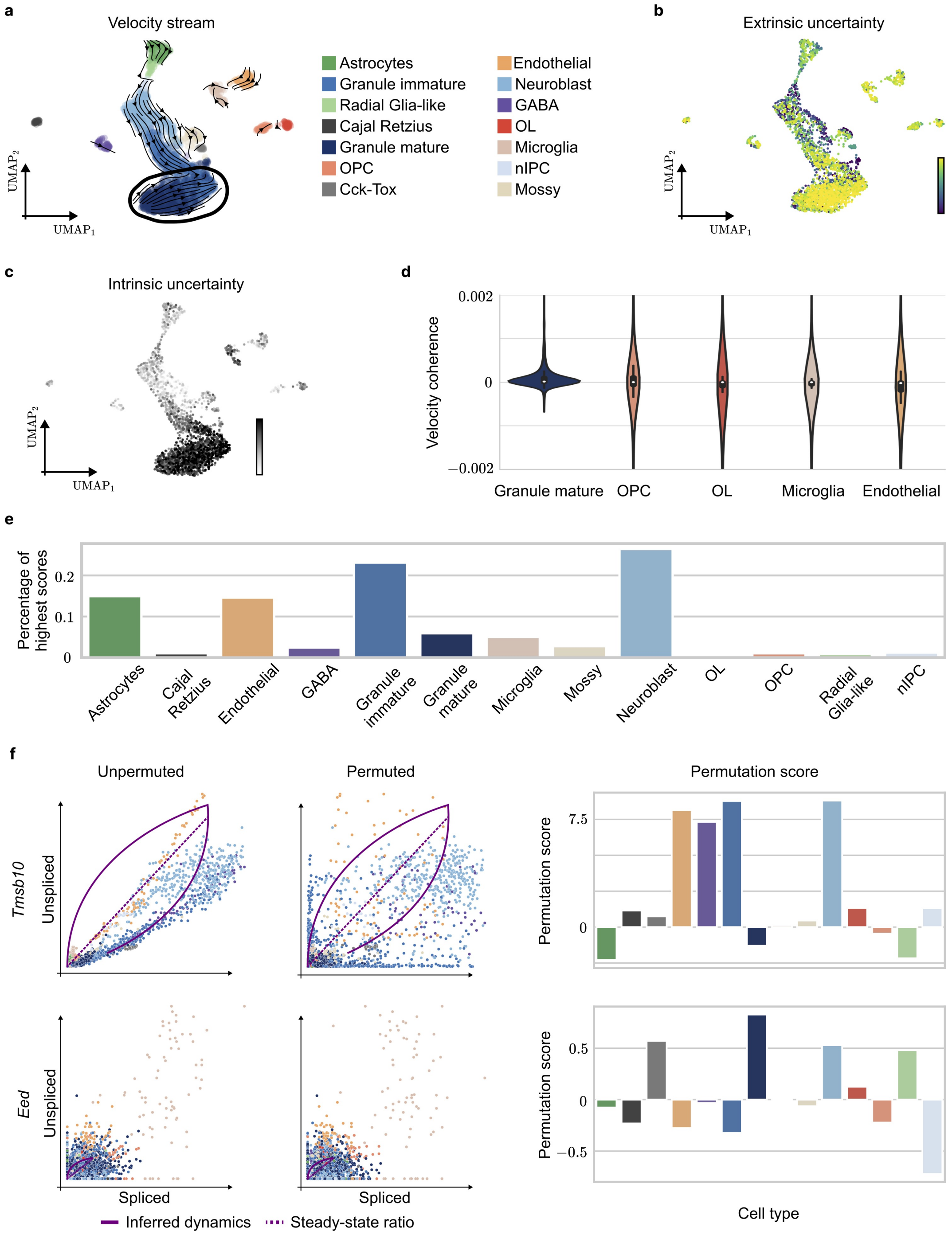

Supplementary Figure 11: Analysis of dentate gyrus. **a.** Two-dimensional UMAP representation of a dentate gyrus dataset containing 2930 cells. The corresponding velocity stream is projected onto the embedding and represented by black arrows. Each cell is colored by their cell type (oligodendrocyte progenitor cells (OPC), oligodendrocyte (OL)) according to the original work [5]. **b.** UMAP emebdding colored by extrinsic, **c.** and intrinsic uncertainty. **d.** The density of the corresponding velocity coherence is shown for granule mature cells, OPC, OL, microglia, and endothelial cells. Box plots indicate the median (center line), interquartile range (hinges), and whiskers at 1.5x interquartile range ($N = 120$ astrocytes, $N = 34$ cajal retzius cells, $N = 27$ cck-tox cells, $N = 87$ endothelial cells, $N = 61$ GABA cells, $N = 785$ granule immature cells, $N = 1070$ granule mature cells, $N = 81$ microglia, $N = 75$ mossy cells, $N = 417$ neuroblasts, $N = 50$ oligodendrocytes, $N = 53$ oligodendrocyte progenitor cells, $N = 51$ radial glia-like cells, $N = 19$ nIPCs).**e.** The percentage of each cell type scoring the highest permutation score. **f.** The phase portraits of the unpermuted (left), permuted (middle) and resulting permutation score per cell type are given for *Tmsb10* and *Eed*. Coloring corresponds to cell type as defined in panel a.

Supplementary Note 3

Related work

New approaches have been developed to blend RNA velocity with deep-learning-based representation learning. Here we briefly describe each approach and then relate them to the capabilities of veloVI.

VeloAE VeloAE [7] leverages an autoencoder framework to learn a representation of both spliced and unspliced data that can be used to estimate transcriptional dynamics. This autoencoder makes use of a graph convolutional network (GCN) module to smooth cell representations over a graph induced by standard scRNA-seq methods (e.g., principcal components analysis followed by approximate nearest neighbors) and an attention mechanism for its decoder. RNA velocity is defined in the cell representation space of the model and and as a result has no mechanistic interpretation nor a directly interpretable link to genes.

DeepVelo (GCN-based) DeepVelo [8] seeks to generalize RNA velocity to multi-lineage systems with cell-specific kinetics. To achieve this, DeepVelo leverages GCNs to encode spliced and unspliced abundance for a cell while aggregating over spliced-abundance-induced neighbourhood graph. In the decoding phase, DeepVelo predicts cell-specific α, β, and γ parameters and uses the mechanistic definition of velocity to compute a cell-specific velocity. This velocity is trained to be predictive of future cell states under a first-order approximation and future cell states are restricted to nearest neighbors. This assumption is referred to as the "continuity assumption". As cell neighborhoods will contain future and past cell states under this assumption, DeepVelo adds a loss term that enforces velocity to be correlated (resp., anticorrelated) with unspliced (resp., spliced) abundance. While DeepVelo leverages ideas from RNA velocity, it does not explicitly model time and thus makes use of standard pseudotime procedures based on its outputs. A similar mechanism of using cells from the training data as a proxy to infer future states of a cell has also been proposed in parallel by Marot-Lassauzaie et al.[9].

DeepVelo (Neural-ODE-based) DeepVelo [10] combines representation learning with neural-network-based ordinary differential equations to learn velocity fields. The model leverages denoising variational autoencoders (VAE) to learn a mapping between the spliced abundance and the velocity estimated with scVelo. They further leverage the VAE within external black-box ordinary differential equation solvers to simulate past or future states of each cell.

DeepCycle In contrast to previous approaches, DeepCycle [11] does not estimate RNA velocity parameters. The method exploits the concept of RNA velocity and its connection to cell-cycle to infer a 1-dimensional latent variable representing the cell-cycle phase using an autoencoder framework.

VeloVAE Upon finalizing this manuscript, we came across two independent manuscripts describing VeloVAE [12, 13]. VeloVAE uses a variational autoencoder framework to estimate kinetic parameters and learns a posterior distribution over a cell-specific latent time. The model likelihood makes use of the solution of ordinary differential equations describing RNA metabolism. VeloVAE posits a cell-gene specific transcriptional rate, which is the function of a low-dimensional latent variable (capturing cell state). Uncertainty in the VeloVAE model is quantified by a coefficient of variation on the low-dimensional latent variable.

Relation to veloVI Our approach veloVI exploits the variational inference (VI) [14] framework to infer each cell's latent time and transcriptional latent state via a local low-dimensional latent variable. Compared to the existing methods, veloVI is the only method that directly estimates RNA velocity at the level of a cell and gene via a biophysical model of transcriptional dynamics *and* leverages uncertainty in its estimate of velocity in downstream applications. The closest approaches directly estimating transcriptional parameters α, β, and γ are DeepVelo (GCN-based) [8] and VeloVAE [12, 13]. Yet, DeepVelo (GCN-based) learns these parameters such that they produce velocities that conform to the model's "continuity" assumption, as well as the notion that velocity should be correlated with unspliced abundance while anticorrelated with spliced abundance; therefore, it only retains a loose connection to transcriptional dynamics.

At a high level, VeloVAE is conceptually similar to veloVI – both approaches make use of the solved differential equations in the model likelihood and manifest as variational autoencoders. For both models, velocity is computed as a statistical functional of the variational posterior. However, there are key differences.

Compared to VeloVAE, veloVI offers unique features to aid in RNA velocity analysis: Intrinsic and extrinsic uncertainty of the estimated velocity, velocity coherence, and the permutation score. Quantifying uncertainty at the level of RNA velocity (cell-gene-specific, and then aggregated) allows assessing regions of the transcriptomic manifold where RNA velocity is either well supported (low uncertainty), or further investigation is needed (high uncertainty). This information is complemented by the permutation score that helps identify viable genes and transient cell types. Consequently, the parameter inference can be quantified beyond a visual, low-dimensional representation. The permutation score based analysis, allows, for example, to correctly identify a dataset of peripheral blood mononuclear cells as inappropriate for RNA velocity analysis (Supplementary Note 1).

veloVI considers uncertainty over transcription states (e.g., induction and repression) for parameter inference. Contrastingly, VeloVAE uses a cell-gene specific transcription rate, as well as a cell-specific latent time. While VeloVAE preprint [12] includes model uncertainty, the uncertainty is at the level of cells via the cell-specific latent representation (analog to z_n in veloVI). This cell-specific representation has a complex non-linear relationship to the estimated velocity (via a neural network).

While veloVI offers tools to assess if a given dataset is suitable for RNA velocity analysis, VeloVAE does not. Indeed, false positives persist even with VeloVAE's improvements. For example in Figure 4 of ref. [12], spurious transitions appear on the velocity stream plot in a dataset of human bone marrow mononuclear cells going from Memory B to Naive B cell types, as well as going from CD8 memory to NK cell types (both with low VeloVAE cell state uncertainty; Supplementary Figure 5e of ref. [12]).

Supplementary Note 4

Modeling considerations, limitations, and future directions

In this supplementary note, we discuss various aspects of RNA velocity, the veloVI model, and future modeling opportunities and directions.

Inference of absolute RNA velocity

Accurate estimation of kinetic rate parameters allow biophysical analysis and interpretation of developmental processes. While veloVI estimates the splicing rate constant (β), the degradation rate constant (γ), and the transcription rate (α) for each gene (Supplementary Figure 12), the corresponding estimate of RNA velocity is technically not an absolute quantity. This is due to a scaling unidentifiability of the model parameters related to the unknown maximum time of the system [15]. More precisely, consider a scaling constant $\lambda > 0$, and scaled parameters $\tilde{\alpha} = \lambda\alpha$, $\tilde{\beta} = \lambda\beta$, $\tilde{\gamma} = \lambda\gamma$, and scaled time $\tilde{t} = \frac{t}{\lambda}$. Denote the estimated abundance of unspliced and spliced RNA using the original and scaled parameters with u, s, and $\tilde{u}$, $\tilde{s}$, respectively. Then,

$$\begin{aligned} u(t) &= u_0 e^{-\beta t} + \frac{\alpha}{\beta}(1 - e^{-\beta t}) = u_0 e^{-\lambda\beta\frac{t}{\lambda}} + \frac{\lambda\alpha}{\lambda\beta}(1 - e^{-\lambda\beta\frac{t}{\lambda}}) \\ &= u_0 e^{-\tilde{\beta}\tilde{t}} + \frac{\tilde{\alpha}}{\tilde{\beta}}(1 - e^{-\tilde{\beta}\tilde{t}}) = \tilde{u}(t), \end{aligned} \tag{1}$$

and

$$\begin{aligned} s(t) &= s_0 e^{-\gamma t} + \frac{\alpha}{\gamma}(1 - e^{-\gamma t}) + \frac{\alpha - \beta u_0}{\gamma - \beta}(e^{-\gamma t} - e^{-\beta t}) \\ &= s_0 e^{-\lambda\gamma\frac{t}{\lambda}} + \frac{\lambda\alpha}{\lambda\gamma}(1 - e^{-\lambda\gamma\frac{t}{\lambda}}) + \frac{\lambda\alpha - \lambda\beta u_0}{\lambda\gamma - \lambda\beta}(e^{-\lambda\gamma\frac{t}{\lambda}} - e^{-\lambda\beta\frac{t}{\lambda}}) \\ &= s_0 e^{-\tilde{\gamma}\tilde{t}} + \frac{\tilde{\alpha}}{\tilde{\gamma}}(1 - e^{-\tilde{\gamma}\tilde{t}}) + \frac{\tilde{\alpha} - \tilde{\beta}u_0}{\tilde{\gamma} - \tilde{\beta}}(e^{-\tilde{\gamma}\tilde{t}} - e^{-\tilde{\beta}\tilde{t}}) = \tilde{s}(t). \end{aligned} \tag{2}$$

This ambiguity only affects the magnitude of the velocity vector per cell, not the direction, as each gene is placed on the same time scale. However, as discussed in ref. [9], the common time scale across genes can result in another form of relativity of velocities between genes, many of which in practice may not display full induction and repression dynamics as assumed by the commonly used transcriptional models. One potential solution for this problem is to assume a global time for the cell that is shared across genes; however, this can hamper the ability of models to infer multiple concurrent dynamical processes (e.g., cell cycle and differentiation), and would therefore require more advanced forms of gene selection.

Beyond the aforementioned challenges, another roadblock to accurately estimating absolute RNA velocity from conventional scRNA-seq data is the biased detection of unspliced transcripts, which in turn result in biased velocity estimates. This bias is caused by internal priming events that entail a possible correlation between captured unspliced reads and gene length bias. Such biases might be alleviate by more advanced sequencing protocols such as VASA-seq, a recently developed sequencing protocol to detect the entire transcriptome in single cells [16]. Rigorous benchmarks will be required to verify the benefit of such protocols for RNA velocity analysis, though. In practice, skewed read detection additionally affect which genes have a strong influence on the velocity-induced cell-cell transition matrix.

Finally, we note that while velocities estimated by veloVI are not absolute, the source of relativity is distinct from that in the original formulation of RNA velocity [17]. In the original RNA velocity formulation, the splicing rate constant β was fixed to 1 for each gene. This constraint renders the estimated degradation rate $\tilde{\gamma} = \frac{\gamma}{\beta}$, and velocities v to be relative to the true, but unestimated splicing rate for each gene:

$$v = u - \tilde{\gamma}s. \tag{3}$$

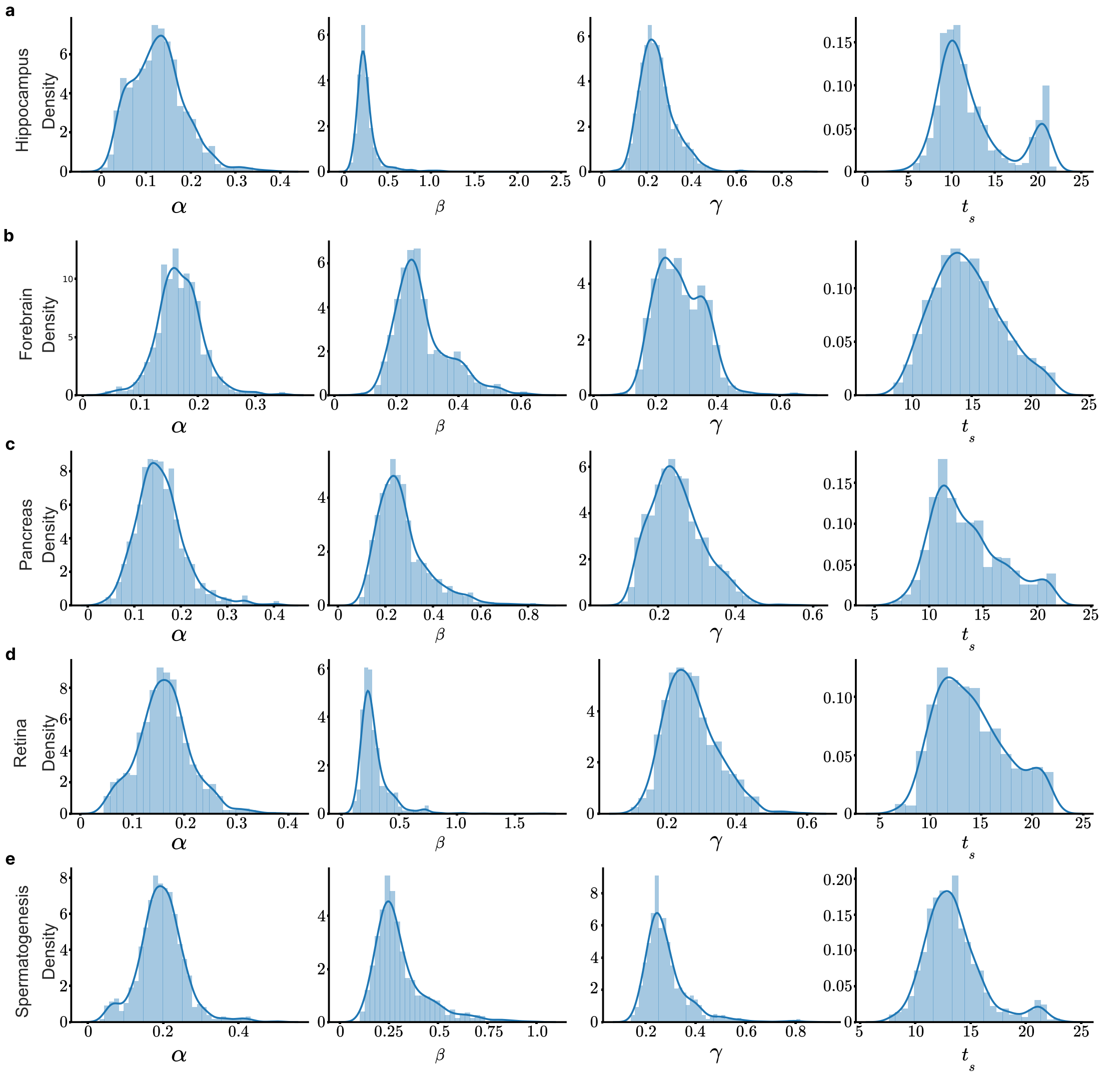

Supplementary Figure 12: Distribution of estimated parameters. The distribution of estimated transcription rate α, splicing rate β, degradation rate γ, and switching time t_s for the **a.** Hippocampus **b.** Forebrain **c.** Pancreas **d.** Retina, and **e.** Spermatogenesis datasets.

Constraints on formulation of rates and rate constants

As in previous work [17], here, we assumed gene-constant transcriptional rates for the mechanistic model describing splicing dynamics. As previously shown, assuming constant rates, the steady state of spliced and unspliced abundance is given by

$$\bar{u}^{(g)}(t_{ng}, k=1) := \lim_{t_{ng}\to\infty} \bar{u}^{(g)}(t_{ng}, k=1) = \frac{\alpha_{g1}}{\beta_g} \tag{4}$$

$$\bar{s}^{(g)}(t_{ng}, k=2) := \lim_{t_{ng}\to\infty} \bar{s}^{(g)}(t_{sg}, k=1) = \frac{\alpha_{g1}}{\gamma_g}. \tag{5}$$

As such, this simplifying assumption guarantees that if the steady-state of the induction phase is observed, it is located in the upper right part of the phase portrait. The closed-form solution of the splicing kinetics used throughout this work is valid under the assumption of constant rates. This assumption is not guaranteed to be valid for non-constant rates. In the case of the time-dependent transcription rate

$$\alpha^{(k)}(t) = \begin{cases} \alpha_1 - (\alpha_1 - \alpha_0)e^{-\lambda_\alpha t}, & k \in \{1,2\}, \\ 0, & k \in \{3,4\}, \end{cases} \tag{6}$$

we fit in this work, the induction steady-state similarly converges to the upper right part of the phase portrait as time tends to infinity.

While cell-specific rates describe real-world biological systems more accurately, a more complex model arises. Considering, for example, cluster-specific rates requires cluster-specific initial conditions, which in turn requires a priori knowledge of the cell lineage. Similarly, a cell-specific transcription rate of arbitrary form no longer allows solving the dynamical system in closed form [18]. As such, while cell-specific rates describe real-world data more accurately, they also entail several adaptions such as the veloVI likelihood. Additionally, as discussed in the previous paragraph, the induction and repression phase can only be assigned to the upper and lower part of the phase portrait, respectively, under certain conditions.

In this work, to achieve cell-specific transcription rates, we relied on tractable time-dependent functional forms for $\alpha(t)$. Future work may explore more flexible function classes, like neural nets, for which the model likelihood would need to be computed using differentiable ordinary differential equation solvers [19].

Model likelihood

In this work, veloVI uses independent mixtures of Gaussians for the likelihood of spliced and unspliced RNA abundances. We anticipate that there are several ways this formulation can be improved in future work.

First, we use the mixture model to account for uncertainty in the state of a cell/gene pair (e.g., induction or repression phase); however, it also helps to make the likelihood differentiable. Indeed, due to the switching time parameter per gene, which is the time in which the positive transcription rate switches to zero, the likelihood is not generally differentiable. Therefore, future work might consider differentiable functions that model transcription rates going to zero as time progresses and eliminating the mixture model, which results in the need to fit many additional parameters.

Second, the Gaussian likelihood does not capture interpretable splicing kinetics [18]. The current formulation also assumes that the data have been smoothed over nearest neighbors, which on one hand justifies the use of a Gaussian under the central limit theorem, but on the other hand, induces a dependence between observations. With the veloVI framework, we expect to study model extensions that leverage count-based likelihoods, such as independent Poisson distributions to model unspliced and spliced counts directly. More complex likelihoods [18] that account for the correlation of unspliced and spliced counts of a gene in one cell should also be explored. Such efforts may also eliminate the biases created when processing the data using commonly used techniques [17, 20].

Exploration with velocity vector fields

While veloVI is trained on a given dataset, unobserved data can be passed to the generative model as well. Consequently, compared to the vector field inference approach proposed in Dynamo [21], no additional step is required to infer vector fields for unobserved data. Thus, future work may explore using the veloVI model in a general way to explore vector fields of transcriptional dynamics. However, while unobserved data can be used in general, for real-world data, the model will also need to be extended to remove batch effects.

Extensions for metabolic labeling data

In this work, we focused on inferring splicing kinetic rates using conventional scRNA-seq data. However, recent technological advances allow metabolically labeling newly produced RNA at scale, and, thereby, measuring its production in a given time frame [22–24]. Consequently, the corresponding datasets hold promise to infer RNA velocity more faithfully due to the increased information content. The recently published approach *Dynamo* has been developed to infer rate parameters for metabolic labeling data [21].

As veloVI is an extensible framework, in future work, we intend to modify the model to infer kinetic rates using metabolically labeled transcripts. Compared to *Dynamo*, this model extension would allow inferring latent time as well as assessing parameter and velocity uncertainty. Additionally, even though *Dynamo* infers degradation and splicing rates γ and β, respectively, it does so in part by relying on the steady-state assumption of the *steady-state model* [17]. An extension of veloVI would no longer make use of this assumption as steady-states are not always observed. Finally, *Dynamo* estimated parameters in a sequential manner (for example, γ from metabolic labels, and β via the steady-state ratio

$\tilde{\gamma} = \frac{\gamma}{\beta}$). Contrastingly, parameters would be estimated end-to-end in a single step by the updated veloVI framework.

Supplementary Tables

Dataset	Reference	Organism	Number of observations
Pancreatic endocrinogenesis	[25]	Mouse	3,696
Spermatogenesis	[26, 27]	Mouse	1,829
Hippocampus	[17]	Mouse	18,213
Forebrain	[17]	Human	1,720
Retina	[28, 29]	Mouse	2,726
Brain	[27, 30]	Mouse	1,823
Prefrontal cortex	[27, 31]	Mouse	1,267
PBMC	[1]	Human	11,950
Dentate gyrus neurogenesis	[5]	Mouse	2,930
Retina (Runtime analysis only)	[32]	Mouse	113,909
RPE1-FUCCI cells	[23]	Human	2,793
U2OS-FUCCI cells	[33]	Human	1,146

Supplementary Table 1: Overview of datasets used in this manuscript.

References

1. Genomics, 1. *10k PBMCs from a healthy donor, Single Cell Gene Expression Dataset by Cell Ranger 6.1.0* 2021.
2. Stuart, T., Butler, A., Hoffman, P., Hafemeister, C., Papalexi, E., Mauck III, W. M., Hao, Y., Stoeckius, M., Smibert, P., *et al.* Comprehensive integration of single-cell data. *Cell* (2019).
3. Melsted, P., Ntranos, V. & Pachter, L. The barcode, UMI, set format and BUStools. *Bioinformatics* (ed Birol, I.) (2019).
4. Gayoso, A., Steier, Z., Lopez, R., Regier, J., Nazor, K. L., Streets, A. & Yosef, N. Joint probabilistic modeling of single-cell multi-omic data with totalVI. *Nature Methods* (2021).
5. Hochgerner, H., Zeisel, A., Lönnerberg, P. & Linnarsson, S. Conserved properties of dentate gyrus neurogenesis across postnatal development revealed by single-cell RNA sequencing. *Nature Neuroscience* (2018).
6. McInnes, L., Healy, J. & Melville, J. UMAP: Uniform manifold approximation and projection for dimension reduction. *Journal of Open Source Software* (2018).
7. Qiao, C. & Huang, Y. Representation learning of RNA velocity reveals robust cell transitions. *Proceedings of the National Academy of Sciences* (2021).
8. Cui, H., Maan, H. & Wang, B. DeepVelo: Deep Learning extends RNA velocity to multi-lineage systems with cell-specific kinetics. *bioRxiv* (2022).
9. Marot-Lassauzaie, V., Bouman, B. J., Donaghy, F. D., Demerdash, Y., Essers, M. A. G. & Haghverdi, L. Towards reliable quantification of cell state velocities. *PLoS Computational Biology* (2022).
10. Chen, Z., King, W. C., Hwang, A., Gerstein, M. & Zhang, J. DeepVelo: Single-cell transcriptomic deep velocity field learning with neural ordinary differential equations. *Science Advances* (2022).
11. Riba, A., Oravecz, A., Durik, M., Jiménez, S., Alunni, V., Cerciat, M., Jung, M., Keime, C., Keyes, W. M., *et al.* Cell cycle gene regulation dynamics revealed by RNA velocity and deep-learning. *Nature communications* (2022).
12. Gu, Y., Blaauw, D. & Welch, J. D. Bayesian Inference of RNA Velocity from Multi-Lineage Single-Cell Data. *bioRxiv* (2022).
13. Gu, Y., Blaauw, D. T. & Welch, J. *Variational Mixtures of ODEs for Inferring Cellular Gene Expression Dynamics* in *International Conference on Machine Learning* (2022).
14. Blei, D. M., Kucukelbir, A. & McAuliffe, J. D. Variational inference: A review for statisticians. *Journal of the American statistical Association* (2017).
15. Li, T., Shi, J., Wu, Y. & Zhou, P. On the Mathematics of RNA Velocity I: Theoretical Analysis. *CSIAM Transactions on Applied Mathematics* (2021).
16. Salmen, F., Jonghe, J. D., Kaminski, T. S., Alemany, A., Parada, G. E., Verity-Legg, J., Yanagida, A., Kohler, T. N., Battich, N., *et al.* High-throughput total RNA sequencing in single cells using VASA-seq. *Nature Biotechnology* (2022).
17. Manno, G. L., Soldatov, R., Zeisel, A., Braun, E., Hochgerner, H., Petukhov, V., Lidschreiber, K., Kastriti, M. E., Lönnerberg, P., *et al.* RNA velocity of single cells. *Nature* (2018).
18. Gorin, G., Fang, M., Chari, T. & Pachter, L. RNA velocity unraveled. *PLOS Computational Biology* (2022).
19. Chen, R. T. Q., Rubanova, Y., Bettencourt, J. & Duvenaud, D. Neural Ordinary Differential Equations. *Advances in Neural Information Processing Systems* (2018).
20. Bergen, V., Lange, M., Peidli, S., Wolf, F. A. & Theis, F. J. Generalizing RNA velocity to transient cell states through dynamical modeling. *Nature Biotechnology* (2020).
21. Qiu, X., Zhang, Y., Martin-Rufino, J. D., Weng, C., Hosseinzadeh, S., Yang, D., Pogson, A. N., Hein, M. Y., Min, K. H. (, *et al.* Mapping transcriptomic vector fields of single cells. *Cell* (2022).
22. Erhard, F., Baptista, M. A. P., Krammer, T., Hennig, T., Lange, M., Arampatzi, P., Jürges, C. S., Theis, F. J., Saliba, A.-E., *et al.* scSLAM-seq reveals core features of transcription dynamics in single cells. *Nature* (2019).
23. Battich, N., Beumer, J., de Barbanson, B., Krenning, L., Baron, C. S., Tanenbaum, M. E., Clevers, H. & van Oudenaarden, A. Sequencing metabolically labeled transcripts in single cells reveals mRNA turnover strategies. *Science* (2020).
24. Qiu, Q., Hu, P., Qiu, X., Govek, K. W., Cámara, P. G. & Wu, H. Massively parallel and time-resolved RNA sequencing in single cells with scNT-seq. *Nature Methods* (2020).
25. Bastidas-Ponce, A., Tritschler, S., Dony, L., Scheibner, K., Tarquis-Medina, M., Salinno, C., Schirge, S., Burtscher, I., Böttcher, A., *et al.* Massive single-cell mRNA profiling reveals a detailed roadmap for pancreatic endocrinogenesis. *Development* (2019).
26. Hermann, B. P., Cheng, K., Singh, A., Cruz, L. R.-D. L., Mutoji, K. N., Chen, I.-C., Gildersleeve, H., Lehle, J. D., Mayo, M., *et al.* The Mammalian Spermatogenesis Single-Cell Transcriptome, from Spermatogonial Stem Cells to Spermatids. *Cell Reports* (2018).
27. Soneson, C., Srivastava, A., Patro, R. & Stadler, M. B. Preprocessing choices affect RNA velocity results for droplet scRNA-seq data. *PLOS Computational Biology* (2021).

28. Giudice, Q. L., Leleu, M., Manno, G. L. & Fabre, P. J. Single-cell transcriptional logic of cell-fate specification and axon guidance in early born retinal neurons. *Development* (2019).
29. Kharchenko, P. V. The triumphs and limitations of computational methods for scRNA-seq. *Nature Methods* (2021).
30. Ximerakis, M., Lipnick, S. L., Innes, B. T., Simmons, S. K., Adiconis, X., Dionne, D., Mayweather, B. A., Nguyen, L., Niziolek, Z., *et al.* Single-cell transcriptomic profiling of the aging mouse brain. *Nature Neuroscience* (2019).
31. Bhattacherjee, A., Djekidel, M. N., Chen, R., Chen, W., Tuesta, L. M. & Zhang, Y. Cell type-specific transcriptional programs in mouse prefrontal cortex during adolescence and addiction. *Nature Communications* (2019).
32. Melsted, P., Booeshaghi, A. S., Liu, L., Gao, F., Lu, L., Min, K. H., da Veiga Beltrame, E., Hjörleifsson, K. E., Gehring, J., *et al.* Modular, efficient and constant-memory single-cell RNA-seq preprocessing. *Nature Biotechnology* (2021).
33. Mahdessian, D., Cesnik, A. J., Gnann, C., Danielsson, F., Stenström, L., Arif, M., Zhang, C., Le, T., Johansson, F., *et al.* Spatiotemporal dissection of the cell cycle with single-cell proteogenomics. *Nature* (2021).
