# Supplementary material for: Deep generative modeling of transcriptional dynamics for RNA velocity analysis in single cells
Source: Nat Methods. 2023 Sep 21;21(1):50–9. doi: 10.1038/s41592-023-01994-w (PMC10776389; doi:10.1038/s41592-023-01994-w)
Supplement: Supplementary file 2 — Reporting Summary [file 41592_2023_1994_MOESM2_ESM.pdf]

nature portfolio

Corresponding author(s): Theis and Yosef

Last updated by author(s): Apr 13, 2023

Reporting Summary

Nature Portfolio wishes to improve the reproducibility of the work that we publish. This form provides structure for consistency and transparency in reporting. For further information on Nature Portfolio policies, see our Editorial Policies and the Editorial Policy Checklist.

Statistics

For all statistical analyses, confirm that the following items are present in the figure legend, table legend, main text, or Methods section.

n/a	Confirmed	
☐	☒	The exact sample size (*n*) for each experimental group/condition, given as a discrete number and unit of measurement
☐	☒	A statement on whether measurements were taken from distinct samples or whether the same sample was measured repeatedly
☐	☒	The statistical test(s) used AND whether they are one- or two-sided<br>*Only common tests should be described solely by name; describe more complex techniques in the Methods section.*
☐	☒	A description of all covariates tested
☐	☒	A description of any assumptions or corrections, such as tests of normality and adjustment for multiple comparisons
☐	☒	A full description of the statistical parameters including central tendency (e.g. means) or other basic estimates (e.g. regression coefficient) AND variation (e.g. standard deviation) or associated estimates of uncertainty (e.g. confidence intervals)
☐	☒	For null hypothesis testing, the test statistic (e.g. *F*, *t*, *r*) with confidence intervals, effect sizes, degrees of freedom and *P* value noted<br>*Give P values as exact values whenever suitable.*
☐	☒	For Bayesian analysis, information on the choice of priors and Markov chain Monte Carlo settings
☒	☐	For hierarchical and complex designs, identification of the appropriate level for tests and full reporting of outcomes
☐	☒	Estimates of effect sizes (e.g. Cohen's *d*, Pearson's *r*), indicating how they were calculated

Our web collection on statistics for biologists contains articles on many of the points above.

Software and code

Policy information about availability of computer code

Data collection: No software was used.

Data analysis:

anndata 0.8.0
scanpy 1.9.1
h5py 3.7.0
igraph 0.9.11
joblib 1.1.0
leidenalg 0.8.10
llvmlite 0.38.1
loompy 3.0.7
louvain 0.7.1
matplotlib 3.5.2
numba 0.55.2
numpy 1.22.4
pandas 1.3.5
scipy 1.8.1
scvi-tools 0.16.4
sklearn 1.1.1
scvelo 0.2.5.dev71+g85295fc
velovi 0.1.0

veloVI is implemented in a standalone package at https://github.com/YosefLab/velovi, which has also been deposited via Zenodo (https://doi.org/10.5281/zenodo.7897641). Code to reproduce the results in the manuscript can be found at: https://github.com/YosefLab/velovi_reproducibility, as well as deposited via Zenodo (https://doi.org/10.5281/zenodo. 7931042)

For manuscripts utilizing custom algorithms or software that are central to the research but not yet described in published literature, software must be made available to editors and reviewers. We strongly encourage code deposition in a community repository (e.g. GitHub). See the Nature Portfolio guidelines for submitting code & software for further information.

Data

Policy information about availability of data

All manuscripts must include a data availability statement. This statement should provide the following information, where applicable:

- Accession codes, unique identifiers, or web links for publicly available datasets
- A description of any restrictions on data availability
- For clinical datasets or third party data, please ensure that the statement adheres to our policy

The processed Pancreas data, including spliced and unspliced count abundances, can be downloaded from scVelo's GitHub (https://github.com/theislab/scvelo_notebooks/raw/master/data/Pancreas/endocrinogenesis_day15.h5ad). The forebrain and dentate gyrus datasets can be downloaded from the Kharchenko lab at Harvard (forebrain: http://pklab.med.harvard.edu/velocyto/DG1/10X43_1.loom, dentate gyrus: http://pklab.med.harvard.edu/velocyto/hgForebrainGlut/hgForebrainGlut.loom). The Friedrich Miescher Institute for Biomedical Research (https://www.fmi.ch/groups/gbioinfo/RNAVeloQuant/RNAVeloQuant.html) provides the processed data of the dentate gyrus, mouse brain, pancreas, prefrontal cortex, and spermatogenesis. The mouse retina and PBMC data is available for download via figshare (https://figshare.com/projects/veloVI_datasets/145476).

Human research participants

Policy information about studies involving human research participants and Sex and Gender in Research.

Reporting on sex and gender	N/A
Population characteristics	N/A
Recruitment	N/A
Ethics oversight	N/A

Note that full information on the approval of the study protocol must also be provided in the manuscript.

Field-specific reporting

Please select the one below that is the best fit for your research. If you are not sure, read the appropriate sections before making your selection.

☒ Life sciences ☐ Behavioural & social sciences ☐ Ecological, evolutionary & environmental sciences

For a reference copy of the document with all sections, see nature.com/documents/nr-reporting-summary-flat.pdf

Life sciences study design

All studies must disclose on these points even when the disclosure is negative.

Sample size	No sample size calculation was performed. Sample sizes were determined based on preprocessed scRNA-seq data.
Data exclusions	No data were excluded from the analysis.
Replication	All of the findings reported in this study are reproducible based on code in the veloVI reproducibility GitHub repository (code availability).
Randomization	Randomization was not relevant to this study.
Blinding	Blinding was not relevant to this study as there were no case-control comparisons made.

Reporting for specific materials, systems and methods

We require information from authors about some types of materials, experimental systems and methods used in many studies. Here, indicate whether each material, system or method listed is relevant to your study. If you are not sure if a list item applies to your research, read the appropriate section before selecting a response.

Materials & experimental systems

n/a	Involved in the study
☒	☐ Antibodies
☒	☐ Eukaryotic cell lines
☒	☐ Palaeontology and archaeology
☒	☐ Animals and other organisms
☒	☐ Clinical data
☒	☐ Dual use research of concern

Methods

n/a	Involved in the study
☒	☐ ChIP-seq
☒	☐ Flow cytometry
☒	☐ MRI-based neuroimaging
